# American Medical Association

**Published:** 1853-09

**Authors:** 


*American Medical Association.*—At a meeting of the Association, held at New York, in May, 1853, the undersigned were appointed a Committee to receive voluntary communications on medical subjects, and to award two prizes of $100 each, to the authors of the best two essays.

Each communication must be accompanied by a sealed packet, containing the name of the author, which will be opened only in the case of the successful competitors. Unsuccessful communications will be returned on application after June 1st, 1854.

Communications must be addressed, post-paid, to the chairman of the committee, Dr. Charles A. Pope, 123 Locust street, St. Louis, Mo., on or before the 30th of March, 1854.

Charles A. Pope, M. D., Thomas Reyburn, M. D., John S. Moore, M. D., John B. Johnson, M. D., A. Litton, M. D., *Committee.*

CHARLES A. POPE, M. D., *Chairman.*

The medical press of the United States are requested to copy the above notice.

